# A Novel Role of Ascorbic Acid in Anti-Inflammatory Pathway and ROS Generation in HEMA Treated Dental Pulp Stem Cells

**DOI:** 10.3390/ma13010130

**Published:** 2019-12-27

**Authors:** Francesca Diomede, Guya Diletta Marconi, Simone Guarnieri, Michele D’Attilio, Marcos F. X. B. Cavalcanti, Maria A. Mariggiò, Jacopo Pizzicannella, Oriana Trubiani

**Affiliations:** 1 Department of Medical, Oral and Biotechnological Sciences, University “G. D’Annunzio”, Chieti-Pescara, 66100 Chieti, Italy; francesca.diomede@unich.it (F.D.); guya.marconi@unich.it (G.D.M.); michele.dattilio@unich.it (M.D.); 2 Department of Neuroscience, Imaging and Clinical Sciences, University “G. D’Annunzio”, Chieti-Pescara, 66100 Chieti, Italymariggio@unich.it (M.A.M.); 3 Center on Aging Science and Translational Medicine (Ce.S.I.-Me.T.), University “G. D’Annunzio”, Chieti-Pescara, 66100 Chieti, Italy; 4 Biophotonics Laboratory, Nove de Julho University, São Paulo 01506-000, Brazil; 5 Dental Clinic, Nove de Julho University, São Paulo 01506-000, Brazil; 6 ASL 02 Lanciano-Vasto Chieti, « Ss. Annunziata » Hospital, 66100 Chieti, Italy; jacopo.pizzicannella@unich.it

**Keywords:** human dental pulp stem cells, hema, ascorbic acid, nfkb, erk, perk, inflammatory cytokines

## Abstract

Resin (co)monomers issued from restorative dental materials are able to distribute in the dental pulp or the gingiva, to get to the saliva and to the flowing blood. Many authors have recently shown that methacrylate-based resins, in particular 2-hydroxyethylmethacrylate (HEMA), are responsible of inflammatory and autophagic processes in human dental pulp stem cells (hDPSCs) while ascorbic acid (AS), an antioxidant molecule, can assume a protective role in cell homeostasis. The purpose of the current work was to study if 50 µg/mL AS can affect the inflammatory status induced by 2 mM HEMA in hDPSCs, a tissue–specific cell population. Cell proliferation, cytokine release, morphological arrangement and reactive oxygen species (ROS) formation were determined respectively by MTT, ELISA, morphological analysis and dichlorofluorescein assay. The hDPSCs exposed to HEMA let to an increment of ROS formation and in the expression of high levels of inflammatory mediators such as nuclear factor-κB (NFkB), inflammatory cytokines such as interleukin IL6, IL8, interferon (IFN)ɣ and monocyte chemoattractant protein (MCP)1. Moreover, HEMA induced the up-regulation of pospho-extracellular signal–regulated kinases (pERK)/ERK signaling pathway associated to the nuclear translocation. AS treatment significantly down-regulated the levels of pro-inflammatory mediators. Then, the natural product AS reduced the detrimental result promoted by methacrylates in clinical dentistry, in fact restore cell proliferation, reduce the pro-inflammatory cytokine, downregulate ROS production and of NFkB/pERK/ERK signaling path. In synthesis, AS, could improve the quality of dental care and play a strategic role as innovative endodontic compound easy to use and with reasonable cost.

## 1. Introduction

Methacrylate-based dental resins are utilized in the clinic for their reliable aesthetic and functional properties. Earlier studies have describedthat the (co)-monomers tri-ethylene glycol dimethacrylate (TEGDMA) and 2-hydroxyethyl methacrylate (HEMA) can be processed into intermediate compounds as epoxy metabolites 2,3-epoxy-2-methyl-propionicacid-methylester, 2,3-epoxy-2-methylpropionic acid and methacrylic acid [1,2,3,4]. In clinical practice, methacrylate-based dental resins could present some critical issues. Indeed, during the polymerization the free (co)monomers can take contact with oral mucosa and throughout dentinal tubules, with dental pulp tissue but also, then also reaching the vascular system various organs inducing an inflammatory status [5,6]. It has been widely described in the literature that HEMA evokes different inflammatory responses depending on its concentrations and on the specific cell-type stimulated [7,8]. In addition, the nuclear factor-κB (NFkB) pathway, a family of ubiquitously expressed transcription factors regulating the cell response to stress, has been shown as a possible key molecule in the HEMA-modulated intracellular pathways [9,10,11].

Based of the literature, resin materials, such as HEMA, are applied in several dental restorative procedures such as surface sealers, cementation of intraradicular posts and repair of restoration. However, as largely reported in the literature, the dental composite and the materials characteristics represents just one portion of a complicated issue. In fact, the accomplishment of clinical restorations determines by a range of components containing accurate technique, suitable materials and precise patient assortment [12].

Evidences show that pulp tissues in vivo have a scarce restorative ability in the proximity of dentin adhesives [13,14]. This advises that a straight interaction between the dentin adhesives, with their constituents, and pulp tissues guides to negative outcomes on pulp injury recovery. Indeed, despite the growing popularity of the use of these adhesives, there are concerns about HEMA, one of the main component (constituting about 30–50%) of dentin adhesives, possible toxicity, based on the fact that it may be released by resin restorations [7,15,16]. 

In dental pulp stem cells (hDPSCs), low concentrations of HEMA (3 and 5 mmol L^−1^) promote an increase of pro-inflammatory mediators, as IL6, IL8, and MCP1 proteins and the consequentactuation of signaling pathway NFkB/ERK/pERK [17,18], and can also activate cell autophagy as pro-survival cytoprotective process to restore cell homeostasis [19]. Autophagy machinery uses the lysosomal activities in health and disease control. In particular mitophagy, a selective autophagy system, encourages the mitochondria breakdown in reaction to injury or stress. Impaired mitochondria are able to promote high levels of reactive oxygen species (ROS) [20]. ROS contain: superoxide anion, hydrogen peroxide and hydroxyl radical, thus representing a class of important signaling and mediator molecules skilled to regulate multiple biological processes [21,22].

Medium levels of ROS are crucial in physiological cellular processes, while high degrees of ROS induce oxidative stress [23]. Increased level of ROS could be responsible for ischemia–reperfusion injury [24], drug-induced toxicity [25], chronic inflammation [26] and, in addition, responsible of systemic diseases as: cardiovascular, cancer and neurodegenerative diseases [27]. It has been demonstrated that monomers derived from dental resins influencing the cell oxidative machinery induced an increase of intracellular ROS in many cell types including dental pulp cells [28,29,30,31].

Nowadays, there is a growing interest in the development of alternative natural molecules to counteract an induced-oxidative unbalance. Ascorbic acid (AS) is an important water-soluble vitamin for humans and other species. It is a critical antioxidant factor that functions as a cofactor in several enzymes’ activity and its deficiency was found involved in many pathological conditions [32,33]. This molecule should be incorporated in the diet to assure tissue homeostasis, because persons have misplaced the capability to synthesize ascorbate for the growth of mutations in the coding sequence of the L-gulono-1,4-lactone oxidase [34,35,36]. Indeed, AS have been widely used in the treatment of many pathological conditions, including common cold, diabetes, cataracts, glaucoma, heart disease, and even cancer [37,38,39]. AS is also considered a crucial antioxidant and cofactor, which is implicated in the healthy growth, task and preservation of several cell types in the body, such as: adipocytes, myoblast, chondroblasts, odontoblasts and osteoblasts [40,41,42]. In dental practice, this essential cofactor, AS, has been integrated into the commercial composites Venus®, Grandio® and FiltekTM Supreme XTE. Yangand and Coll. have evaluated the effects of AS presence on the rate of transformation and elution of composite elements from the above mentioned composites. It was hypnotized that the significant reduction of degree of conversion in AS-containing composites can be due to the antioxidant properties of AS itself [3].

Starting from these considerations the intention of this work was to assess the potential protective effect of AS on hDPSCs treated with 2 mM HEMA, and to define its possible mechanism. This hypothesized effect could have a positive impact on cell homeostasis during dental care applications.

## 2. Materials and Methods

### 2.1. Ethic Statement

The current work was accepted by the Ethical Committee at the Medical School, “G. d’Annunzio” University, Chieti, Italy (number 266/April 17, 2014). Informed consent was assigned by all registered subjects. 

### 2.2. Cell Culture Establishment

Human DPSCs were taken from the dental pulp of non-carious third molars removed for orthodontic aim, as previously described [43]. Dental pulp tissue fragments were placed in a culture dish with mesenchymal stem cell growth medium - chemically defined

(MSCGM-CD, Lonza, Basel, Switzerland) and were cultured in an incubator at 37 °C in a humidified atmosphere of 5% CO_2_ in air, as earlier reported [44].

### 2.3. Cell Culture Establishment 

Characterization of hPDLSCs were established by cytofluorimetric analysis as earlier reported [19]. Briefly, hPDLSCs were evaluated for the presence or absence of Sox-2, Oct3/4, CD13, C14, CD29, CD34, CD45, CD73, CD90, and CD105. The following conjugated antibodies were utilized: fluorescein isothiocyanate anti-CD13 (CD13 FITC), phycoerythrin anti-CD29 (CD29 PE), FITC anti-CD45 (CD45 FITC), and anti-CD105 (CD105 FITC), were acquired from Ancell; FITC anti-CD14 (CD14 FITC) was purchased from MiltenyBiotec; PE anti-CD73 (CD73 PE), FITC anti-CD90 (CD90 FITC), Alexa 488 anti-Sox2 (Sox2 Alexa 488), FITC anti-SSEA-4 (SSEA-4 FITC), and PE anti-OCT3/4 (OCT3/4 PE) purchased from Becton Dickinson; PE anti-CD34 (CD34-PE) was obtained from Beckman Coulter; as secondary antibody was used a FITC-conjugated achieved from Jackson Immunoresearch Laboratories. As washing buffer (WB) a PBS solution consisting of 0.1% sodium azide, and 0.5% bovine serum albumin was utilized. Samples (5 × 10^5^ cells) were incubated with 100 mL of 20 mM EDTA at 37 °C for 10 min and then washed. Staining of the antigens were performed according to Trubiani et al. [44]. As routine control was used the rainbow calibration particles (BD Biosciences). Debris was omitted from the measurements by gating on morphological parameters; for each sample, at least 20,000 events were recorded. All antibodies were titrated under assay conditions, and optimal photomultiplier (PMT) gains were found for each channel. Data were measured by means of FlowJo software (TreeStar). Mean fluorescence intensity ratio (MFI Ratio) was evidenced by the ratio of the MFI positive and the MFI negative occurences.

Cell morphology was determined according to Gugliandolo et Coll. fixing and stain the hPDLSCs using toluidine blue solution Images were captured by an inverted Leica DMIL light microscopy equipped with Leica EC3 camera [43].

Osteogenic differentiation, was assessed using colture media kit from Lonza (Basel, Swisse). After 21 days in differentiation media, the cells were dyed for Alizarin red S solution. For adipogenic differentiation, hPDLSCs were cultured in adipogenic medium kit (Lonza) for 28 days. At the end of induction time, the cells were stained utilizing Adipo Oil red O solution [45].

Real-time PCR was also performed to investigate the expression of runt-related transcription factor 2 (RUNX2), alkaline phosphatase (ALP) and fatty acid binding protein-4 (FABP4), peroxisome proliferator-activated receptor-ɣ (PPARɣ) to confirm the osteogenic and adipogenic differentiation respectively [46].

Total RNA was obtained from osteogenic and adipogenic differentiated or control hDPSCs using the RNeasy Plus Universal Mini Kit (Qiagen, Valencia, CA, USA). The ABI PRISM 7900 HT Sequence Detection System (Applied Biosystems, Foster City, CA, USA) was used for qPCR of studied markers (ALP Hs01029144_m1; RUNX2 Hs00231692_m1; FABP4 Hs01086177_m1; PPARɣ Hs01115513_m; Applied Biosystems, Beta-2 microglobulin (B2M, Hs99999907_m1; Applied Biosystems) was used for template normalization [47]. Comparative 2^−ΔΔCt^ relative quantification method was used to analyze the mRNA expression.

### 2.4. Study Design

Human DPSCs at passage 2 were used for the experiments executed in triplicate. The following experimental groups have been reported:

- Untreated hDPSCs, utilized as negative control (CTRL);
- hDPSCs treated for 24 h with 2 mM HEMA (HEMA);
- hDPSCs treated for 24 h with 50 µg mL^−1^ ascorbic acid (AS);
- hDPSCs co-treated for 24 h with 2 mM HEMA and 50 µgmL^−1^ ascorbic acid (HEMA + AS);
- hDPSCs co-treated for 24 h with 2 mM HEMA and 1 mM N-acetyl Cysteine (HEMA + NAC);

Based on the literature, Knon JH et al., reported that 2 mM of HEMA is the highest non-cytotoxic concentrations to be used to evaluate the cell viability of hDPSCs in response to HEMA; for this reason, it was considered a suitable dose for further investigations [48]. Furthermore, ROS scavenger effect of Ascorbic acid in HEMA treated cells was compared to N-Acetyl-l-cysteine taking into account for the concentration used on the basis of what is already known in the literature [49,50] 

### 2.5. MTT Assay 

Cell viability of treated and untreated hDPSCs was obtained utilizing tetrazolium compound [3-(4,5-dimethylthiazol-2-yl)-5-(3-carboxymethoxyphenyl)-2-(4-sulfophenyl)-2H-tetrazolium, inner salt; MTS]. Human DPSCs (2 × 10^3^ cells/well) were plated in 96-well tissue culture plates and incubated at 37 °C for 24, 48 and 72 h. At each time point, 20 µl of MTT solution (The Cell Titer 96 AQueous One solution reagent, Promega, Milan, Italy) was supplemented to each well to distinguish the metabolic activity of the cells. All plates were incubated in the dark for 3 h at 37 °C and then read at 490 nm wavelength using a microplate reader (Synergy HT, BioTek Instruments, Winooski, VT, USA) [51]. 

### 2.6. Wound Healing Assay

Wound-healing analysis was executed to assess the percentage of wound closure after 24 h-treatments [52]. In brief, hDPSCs were cultured in 60mm-ø plates (3 × 10^5^ cells/plate). After 24 h–seeding, cell monolayers were wounded using a sterile pipette (10 μL tip). To remove cellular debris the medium was replaced using a fresh medium before cell treatment. The percentage of wound closure was evaluated by cellular analysis using an inverted light microscope (DMIL, Leica Microsystem, Milan, Italy) and determined after 8h-and 24h-treatmentby calculating the migrated distance/total wound distance using the LEICA LAS/EZ software (3.4).

### 2.7. Immunohistochemistry and Confocal Laser Scanning Microscope (CLSM) Analysis

The hDPSCs were fixed using 4% paraformaldehyde solution in sodium phosphate buffer (Lonza, Basel, Switzerland) [53,54]. The cells were permeabilized utilizing 0.5% Triton X-100 in PBS for 10 min followed by blocking with 5% skimmed milk in PBS for 30 min [55]; primary antibodies (anti-NFkB antibody, 1:250, Santa Cruz Biotechnology; anti-ERK antibody,1:200, Santa Cruz Biotechnology; anti-pERK antibody,1:200, Santa Cruz Biotechnology) were incubated for 2 h at room temperature. At the end of incubation cells were processed using secondary antibody (Alexa Fluor 568 red fluorescence conjugated goat anti-rabbit antibody, 1:200, Molecular Probes, Invitrogen, Eugene, OR, USA) incubation for 1 h at 37 °C. To depict the cytoskeleton actin, samples were treated with the Alexa Fluor 488 phalloidin green fluorescent conjugate (1:400, Molecular Probes, Eugene, OR, USA) for 1 h [56]. After washings, cells were incubated with TOPRO (1:200, Molecular Probes) for 1 h at 37 °C [57] to stain cell nuclei. Samples were detected using a Zeiss LSM800 confocal system (Zeiss, Jena, Germany). 

### 2.8. Western Blot Analysis

The cell lysates (30 μg) from entirely sample groups were examined as earlier reported [58], fractionated by SDS-PAGE and successively transferred to nitrocellulose membranes utilizing a semidry blotting machine. Membranes were saturated for 60 min at 37 °C in blocking buffer (1 x TBS, 5% milk, 0.05% Tween-20), then incubated overnight at 4 °C in blocking buffer with primary antibodies against NFkB (1:500, Santa Cruz Biotechnology), ERK (1:1000, Santa Cruz Biotechnology), p-ERK (1:750, Santa Cruz Biotechnology) or β-actin (1:1000, Santa Cruz Biotechnology) [59]. After four washes in TBS containing 0.1% Tween-20, sheets were incubated for 30 min at room temperature with peroxidase-conjugated secondary antibody diluted 1:1000 in 1 x TBS, 5% milk, 0.05% Tween-20 [47]. Bands were detected by the ECL method. The level of recovered protein was analyzed using the Bio-Rad Protein Assay (Bio-Rad Laboratories, Hercules, CA, USA) [60].

### 2.9. ROS Measurements

Human DPSCs were seeded in 35 mm-ø dish (µ-Dish, ibidi GmbH, Gräfelfing, Germany) and treated for 24 h in culture medium alone (Control, CTRL) or containing 2 mM HEMA, 50 µgmL^−1^ AS or 2 mM HEMA plus 50 µg mL^−1^ AS HEMA + AS. After the incubation time, the cells were washed two times with normal external solution (NES, containing in mM: 125 NaCl, 5 KCl, 1 MgSO_4_, 1 KH_2_PO_4_, 5.5 glucose, 1 CaCl_2_, 20 HEPES, pH 7.4) and incubated for 30 min at 37 °C with 10 μM of 2’, 7’-dichlorodihydrofluorescein diacetate (H2DCFDA, Thermo Fisher Scientific, Monza, Italy) in NES containing the respective HEMA and AS treatments as above described. Then, the cells were washed twice with NES and observed in NES alone (CTRL) or containing HEMA or AS alone, or HEMA + AS. For each experimental condition, confocal pictures were casually taken utilizing a Zeiss LSM800 confocal system (Carl Zeiss, Jena, Germany), equipped with an inverted microscope Axio-obserber. D1 and an objective W-Plan-Apo 40X/1.3 DIC. Excitation was fixed at 488 nm and emission wavelength setting the filter set over 505–530 nm. The acquisition settings (laser power, photomultiplier gain, pin-hole and offset) were maintained constant among all specimens acquisitions. Off-line image analyses were executed using Fiji distribution of ImageJ software (v. 1.5i.) [61].

### 2.10. Cytokines Evaluation

To assess of IL6, IL8, IFNɣ and MCP1, the Quantikine ELISA Kit (R&D Systems, RLB00, Minneapolis, MN, USA) was utilized in agreement with the manufacturer’s instructions. Cell sample preparation was performed as previously reported [46]. Supernatants were collected from hDPSCs of the different experimental groups after 24 h of incubation.

### 2.11. Statistical Analysis

Statistical evaluation has been performed using GraphPad Prims version 4.0 (Prism4 software, GraphPad, La Jolla, CA, USA) utilizing t-test and ordinary one-way ANOVA pursued by post-hoc Bonferroni’s multiple comparisons tests. Values of *p* < 0.05 were estimated statistically important.

## 3. Results

### 3.1. Characterization of hDPSCs

**Figure 1:**
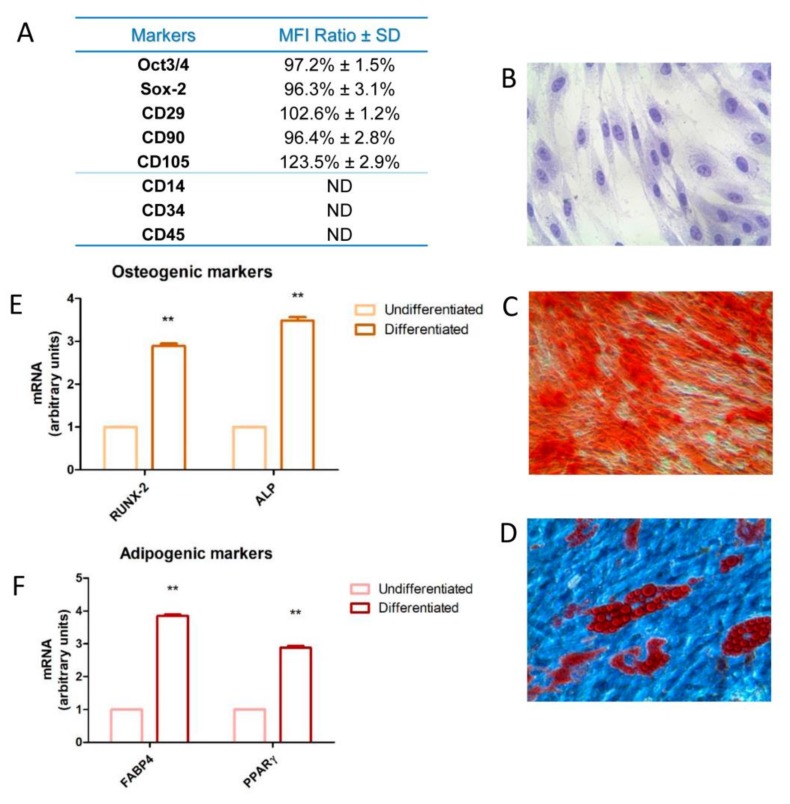
Characterization of human dental pulp stem cells (hDPSC). (**A**) The hDPSCs were analyzed by flow cytometry at second passage. Median fluorescence intensity (MFI) rate is the mean of five different biological samples ± standard deviation (SD); cut off ratio positivity > 2.0. (**B**) Plastic-adherent hDPSCs detected by inverted light microscopy, dyed with toluidine blue staining. (**C**) Osteogenic differentiated hDPSCs dyed with Alizarin Red S solution. (**D**) Adipogenic differentiated hDPSCs dyed with Adipo Oil red O solution. (**E**) Real time-polimerase chain reaction (RT-PCR) of runt-related transcription factor 2 (RUNX2) and alkaline phosphatase (ALP) executed in not differentiated and differentiated hDPSCs. (**F**) RT-PCR of fatty acid-binding protein 4 (FABP4) and peroxisome proliferator-activated receptor gamma (PPARɣ) executed in not differentiated and differentiated hDPSCs. Scale bar: 10 µm. ** *p* < 0.01.

The hDPSCs profiles were analyzed by cytofluorimetric investigations and evaluation of mesengenic differentiation and reported in Figure 1. We found that hDPSCs expressed markers such as Sox-2, Oct3/4, CD29, CD90, and CD105, while they resulted not positive for CD14, CD34, and CD45. Moreover, as shown in Figure 1C,E and in Figure 1D,F, hDPSCs were able to differentiate in osteogenic and adipogenic lineage.

### 3.2. MTT Cell Viability Assay 

**Figure 2:**
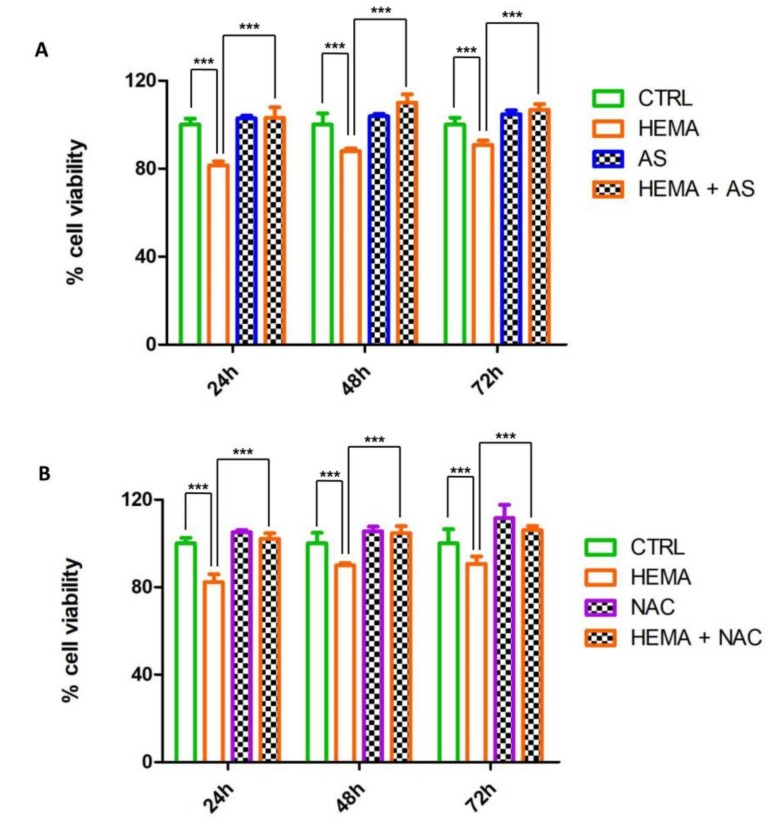
MTT cell viability analysis on hDPSCs. (**A**) Histogram represents the cell viability of hDPSCs exposed to 2 mM HEMA, 50 µg mL^−1^ AS and 2 mM HEMA alone or in co-treatment with 50 µg mL^−1^ AS for 24, 48 and 72 h. HEMA treated cells showed a lower cell viability rate compared to the others samples. The hDPSCs treated with HEMA + AS showed a similar rate to the CTRL group. (**B**) Histogram represents the cell viability of hDPSCs exposed to 2 mM HEMA, 1 mM NAC alone or 2 mM HEMA in co-treatment with NAC for 24, 48 and 72 h. HEMA treated cells evidenced a decrease of cell viability rate respect to the others samples, while hDPSCs administered with HEMA + NAC reported a similar proliferation rate to the CTRL group. The results shown are the average (± SD) of three different analyses. *** HEMA+ AS versus HEMA: *p* < 0.001; *** HEMA versus ctrl: *p* < 0.00 (Figure 2A); *** HEMA + NAC versus HEMA: *p* < 0.001; *** HEMA versus CTRL: *p* < 0.001 (Figure 2B).

To measure the response of HEMA on hDPSCs viable cells, the MTT assay was performed. HEMA (2 mM) alone induced a reduction in the cell viabilityrate. This effect was reverted by the co-presence of AS (50 µg mL^−1^), that did not affect cell proliferation when added alone (Figure 2A). In parallel, it was evaluated the effects of 1 mM of NAC alone or in co-treatment with HEMA on hDPSCs for 24, 48 and 72 h. The presence of NAC evidenced the same positive effects, in terms of proliferation rate, exhibited by AS (50 µg mL^−1^).

### 3.3. HEMA + AS Treatment Produce Migration of hDPSCs

**Figure 3:**
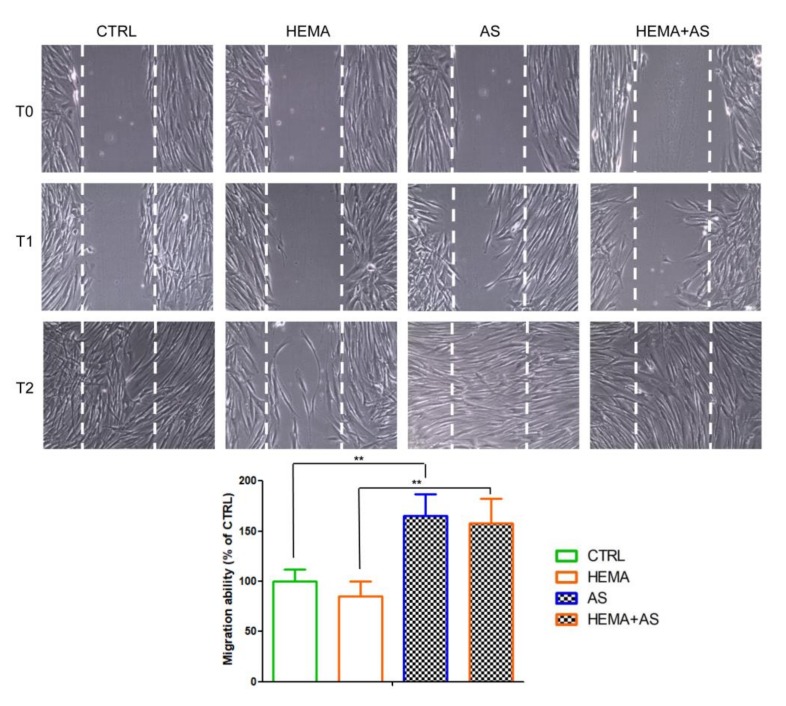
Confluent cell cultures for all conditions were wounded after treatment and migration distances were assessed at T1 (8 h) and T2 (24 h). The results shown in the graph were data from 8 h-treatments reported as percentage of the migration capability towards the control cells and measured using the LEICA LAS/EZ software (3.4) (CTRL, 100%, means ± SD n = 3). The images are representative of three independent experiments. ** HEMA + AS versus HEMA: *p* < 0.01; **AS versus CTRL: *p* < 0.01.

A wound-healing analysis was executed to investigate the ability to migrate of hDPSCs cultured in different conditions. The quantitative analysis was carried out by measuring the migrated distance/total wound space, and expressed as percentage of untreated cells (CTRL). After 8 h-treatment, in AS and HEMA + AS treated cells a significant augmentation of cell migration occurred compared to the other experimental groups. Of note, at 24 h-treatment the healing is not completely closed in HEMA-treated cells, while in the co-presence of AS, it appeared completely closed as well in CTRL and AS-treated cells (Figure 3).

### 3.4. Signaling Pathway ERK, pERK and NF-kB Analyses

**Figure 4:**
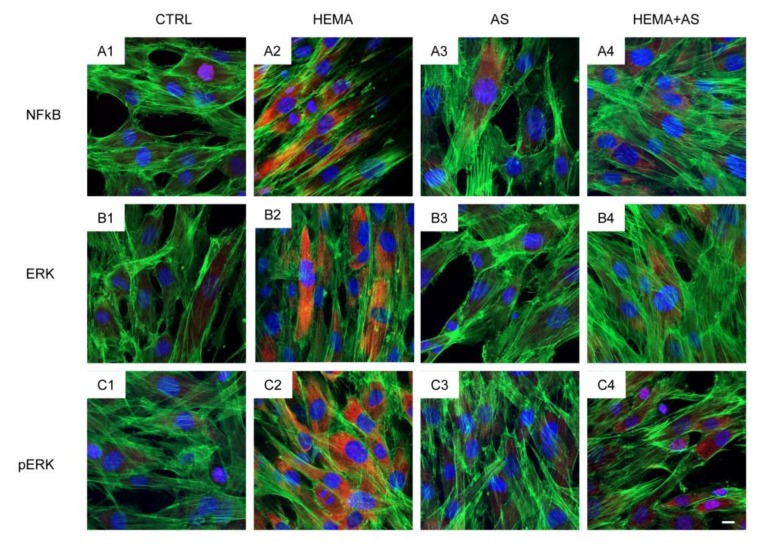
Untreated cells (CTRL) showed a slight fluorescence signal from NFkB- (**A1**), ERK- (**B1**) and pERK-immunostaining (**C1**). Human DPSCs treated with 2 mM HEMA (HEMA) showed an increased fluorescence levels derived from NFkB-(**A2**), ERK- (**B2**) and pERK-immunostaining (**C2**). Cells treated with ascorbic acid (AS) or HEMA + AS showed fluorescence levels similar to CTRL. Green fluorescence derived from Alexa-phalloidin 488 staining for cytoskeleton actin; red fluorescence derived from Alexa Fluor 568-IGg conjugated secondary antibody to reveal primary antibody against NFkB (**A**), ERK (**B**) and pERK (**C**); blue fluorescence derived from TO-PRO staining of nuclei. Scale bar = 10 µm.

Images acquired using a confocal microscope showed the typical fibroblast-like morphology in hDPSCs (Figure 4A1,B1,C1). The HEMA-treated cells (Figure 4A2,B2,C2) showed no evident modifications in the cell morphology respect to control cells (Figure 4A1,B1,C1). The cells treated with 2 mM of HEMA in presence of AS showed a preserving effect, for this reason hDPSCs evidenced a morphology reasonably comparable to the untreated cells (CTRL) (Figure 4A4,B4,C4).

Immunofluorescence experiments to reveal NFkB, ERK and pERK localization were performed in hDPSCs tested in the above mentioned conditions. Pictures in Figure 4 show an increased fluorescence signal derived from NFkB-, ERK- and pERK-immunostaining in HEMA-treated hDPSCs (Figure 4A2,B2,C2), when compared to the hDPSCs in the other experimental conditions, in particular to the cells treated with HEMA + AS. As evidenced in the figure, the cells treated with HEMA exhibited a significant increase of NFkB-, ERK- and pERK expression as evidenced from red fluorescence signal (Figure 4A4,B4,C4).

### 3.5. Western Blot Analyses

**Figure 5:**
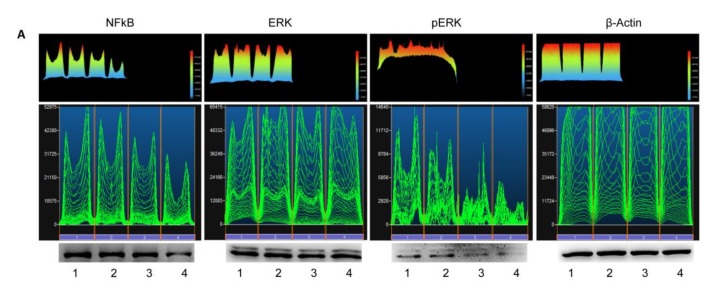

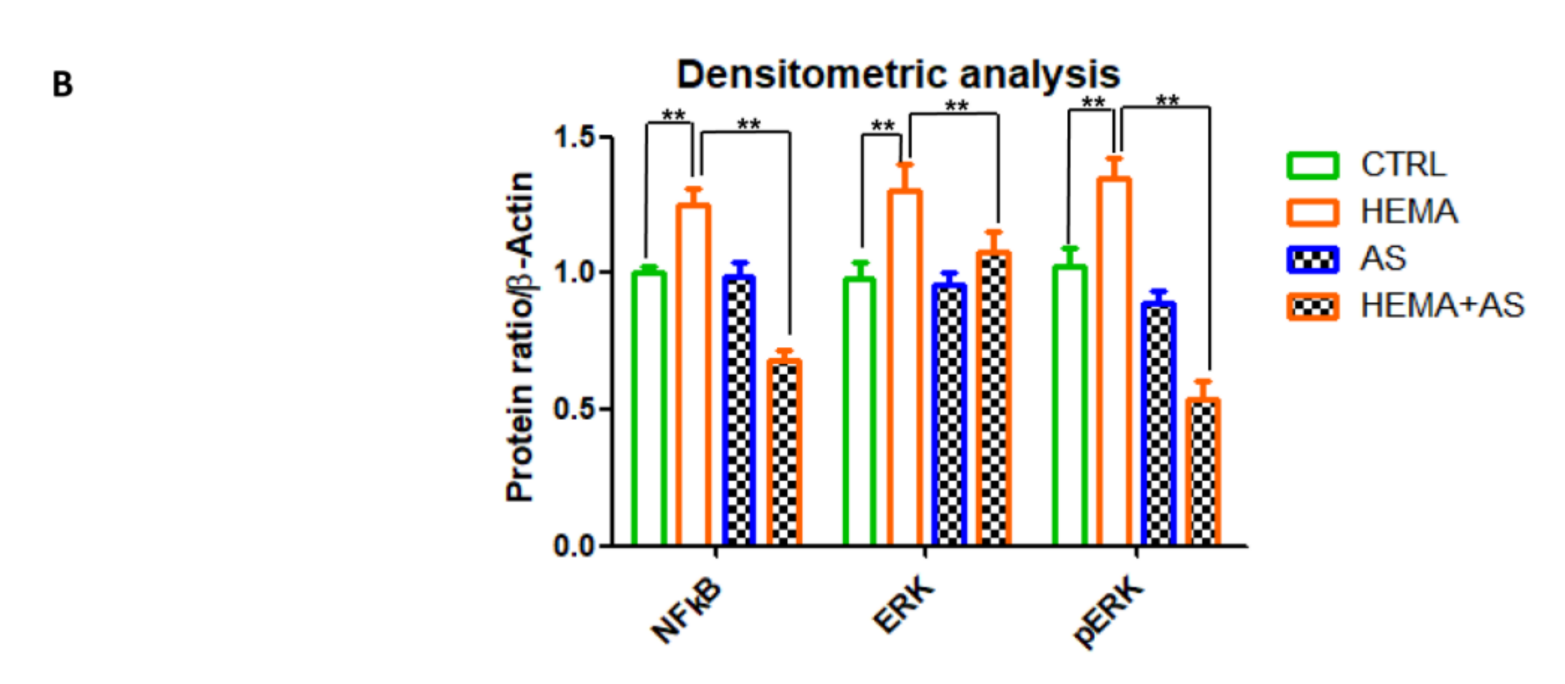
Protein expression. (**A**) Western blotting assay of NFkB, ERK, pERK expression in hDPSCs cells treated with 2 mM of HEMA alone or in presence of AS. Each membrane has been probed with β–actin antibody as housekeeping protein. Western blot is representative of three different analyses. (**B**) Densitometric analysis of proteins bands expressed as ratio of protein quantification normalized with β–actin (mean of three separate experiments). The error bars on these graphs evidence standard deviation (± SD). Graph bars showed the densitometric analysis. ** HEMA + AS versus HEMA: *p* < 0.01; ** AS versus CTRL: *p* < 0.01.

Western blotting assay was executed to evaluate NFkB, ERK and pERK molecule expression. In Figure 5, bands of NFkB, ERK and pERK were increased in HEMA treated in hDPSCs, while in HEMA + AS treated sample, a down regulation of NFkB, ERK and pERK was present (Figure 5).

### 3.6. ROS Production 

**Figure 6:**
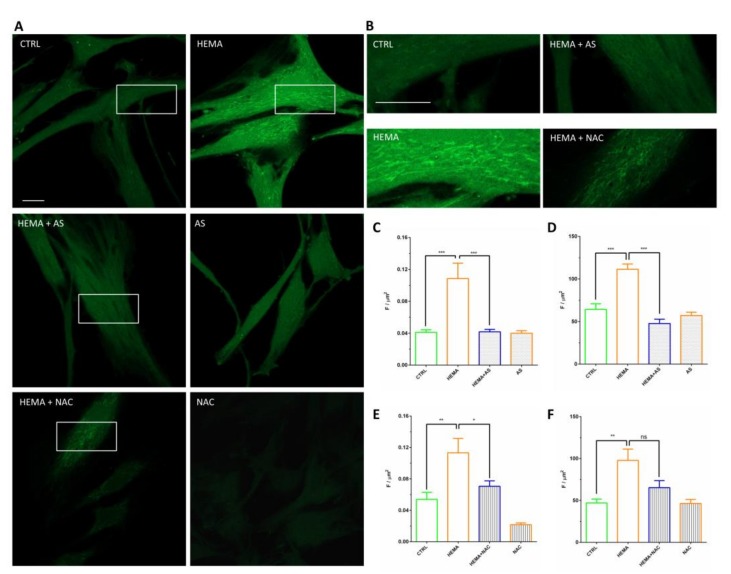
ROS measurements. (**A**) Representative images of live single cells loaded with H2DCFDA, acquired by a confocal microscope: CTRL are untreated cells, HEMA are cells treated with 2 mM HEMA, HEMA + AS are cells treated with 2 mM HEMA plus 50 µg/ml Ascorbic acid, AS are cells treated with 50 µg/ml Ascorbic acid. (**B**) Magnifications of the white boxes present in the above images (CTRL and HEMA; HEMA + AS and HEMA + NAC). (**C**,**E**) graphs of quantitative analysis of ROS production represented as arbitrary unit of fluorescence per cell surface (F/µm^2^). Results are reported as average ± S.E.M (in C: CTRL n = 68, HEMA n = 33, HEMA + AS n = 46, AS n = 42; N = 3; *** *p* < 0.0001; in E: CTRL n = 43, HEMA n = 72, HEMA + NAC n = 74, NAC n = 119; N = 3; ** *p* < 0.001, * *p* < 0.05). (**D**,**F**) Graphs of quantitative analysis of ROS production represented as arbitrary unit of fluorescence per mitochondrial surface (F/µm^2^). Results are evidenced as mean ± S.E.M (in D: CTRL n = 79, HEMA n = 44, HEMA + AS n = 149, AS n = 137; N = 3; *** *p* < 0.0001; in F: CTRL n = 50, HEMA n = 90, HEMA + NAC n = 57, NAC n = 50; N = 3; ** *p* < 0.001). Statistical analysis was executed by one-way ANOVA and post hoc Bonferroni. In panel (**A**,**C**) bar = 20 µm.

To assess the ROS production induced by HEMA, hDPSCs were loaded with the cell-permeant H2DCFDA probe, a chemically reduced form of fluorescein utilized as an indicator for ROS. Confocal microscopy images were acquired on randomly selected fields and the fluorescence recorded was off-line analyzed. In Figure 6A are reported representative images acquired during different experimental conditions. The images revealed a higher fluorescence level in HEMA-treated cells in comparison to all the other experimental conditions (CTRL, AS, HEMA + AS). In addition, while the CTRL sample showed a flat and homogenously distributed fluorescence intensity signal, the HEMA-treated cells showed spaghetti-like area, resembling mitochondria morphology. Interestingly, these peculiar areas in HEMA-treated cells showed higher fluorescence signals than those observed in the other experimental conditions. Quantitative results reported in Figure 6C, derived from off-line analysis of the whole individual cell soma, showed an increase in ROS production in HEMA-treated hDPSCs vs CTRL (means ± S.E.M.: HEMA 0.110 ± 0.012 vs CTRL 0.041 ± 0.003). The co-presence of HEMA and AS appeared to effectively block the ROS increase induced by HEMA (HEMA + AS 0.042 ± 0.003). The AS alone did not modify the ROS basal levels acquired in CTRL samples (AS 0.040 ± 0.003). The quantification performed in the assumed mitochondrial areas (Figure 6D), showed a similar trend among the treatments (HEMA 111.40 ± 6.15 vs CTRL 64.32 ± 6.51; HEMA + AS 47.72 ± 5.00; AS 57.12 ± 4.00). The results from this set of experiments indicate that HEMA treatment showed statistically significant (*p* < 0.0001) higher ROS levels compared with no treated hDPSCs (CTRL), and AS was able to prevent the HEMA-induced ROS production. The AS anti-oxidant ability was compared to that expressed by NAC, a known antioxidant molecule (Figure 6A,B,E). Comparing the images from HEMA + AS treatment *vs* HEMA + NAC treatment it is possible to detect a decreased cytosolic fluorescence signal in both treatments respect to HEMA-treatment, while in images from HEMA + NAC treatment a detectable mitochondrial fluorescence signal still persists (Figure 6B). In our experimental conditions also 1mM NAC is able to prevent HEMA-induced ROS production (HEMA 0.113 ± 0.018 vs HEMA + NAC 0.070 ± 0.007). However, the data derived from the analysis of the mitochondrial areas (Figure 6F) showed that NAC in this district is not able to completely turn off the production of ROS. Indeed, even if NAC-treatment reduced the fluorescence signal, there is no significant difference between the HEMA and HEMA + NAC samples (97.80 ± 13.48 vs. 65.25 ± 8.28).

## 4. Discussion

In this study, for the first time, the effects of HEMA in co-treatment with AS in hDPSCs has been evaluated, in terms of cell viability, cell migration, inflammatory pathways, ROS production and pro-inflammatory cytokines release. HEMA, is one of the most commonly used monomer in dental materials, but it has been reported able to induce anti-proliferative and cytotoxic effects on the hDPSCs, as well as on other cell types [17,19]. It also known that HEMA still presents adverse effects, such as increase in ROS production, concentration-conditioned apoptosis, phosphorylation of extracellular signal-regulated kinase (ERK) [62]. Based on the literature, AS, a well-known antioxidant, plays a pivotal role in physiological and metabolic activities in humans, but it can be only obtained through diet [63]. 

Our data evidenced in hDPSCs treated with 2 mM HEMA a decreased of cell viability starting from 24 up to 72 h, while the co-presence of AS was able to restored the proliferation rate in treated cells, this effect supports the strategic role of this antioxidant molecule in healing/regeneration process [64,65]. In fact, it has been demonstrated that AS promoted wound healing through matrix deposition, neo-vascularization and finally via secretion of inflammatory mediators [66]. For these reasons AS supplementation is used in dentistry to improve postoperative wound healing and in post-surgical dental implant recovery in patients treated with different biomaterials [67].

Mohammed and colleagues showed that AS could promote wound healing process through a variety of mechanisms for example it protects vascular endothelium function through vascular endothelial growth factor (VEGF) upregulation, a fundamental protein in damaged tissue regeneration [68,69]. Furthermore, in our experimental conditions, AS inhibited the HEMA-induced effect on wound healing, accelerating the processes by reducing the scar area in hDPSCs. In this regard, it has been postulated that dental resin monomers trigger an oxidative stress that in turn caused the modulation of mitogen-activated protein kinases (MAPK) signal transduction pathways [70]. Based on this observation, the localization and expression levels of EKR, pERk, and NFkB proteins, as well as ROS production, were assayed in hDPSCs treated with 2 mM HEMA or with HEMA + AS. The results showed that HEMA is able to induce ERK phosphorylation and NFκB translocation, while the co-presence of AS was able to block these effects.

Interestingly, high levels of ROS were found in HEMA-treated hDPSCs after 24 h exposure, this indicated a stable effect of HEMA in ROS production in hDPSCs that did not spontaneously revert. Notably, comparing the confocal fluorescence images acquired in HEMA vs CTRL conditions is clearly evident a higher signal from ROS-sensitive indicator in intracellular area depicting mitochondria structure (in HEMA treated cells, about 1,000 times greater than that calculated from the whole cell soma). This could be due to a persistent HEMA interference into the oxidative phosphorylation as a result of electron transport chain disruption. These data are in agreement with what was recently reported by Jiao and colleagues in a similar cellular model were they observed a depolarization of mitochondrial membrane potential and reduction of oxidative phosphorylation rate [71].

The presence of AS was able to counteract the effect of HEMA, which reduces the generated ROS levels to those of untreated cells. The effect of AS appeared to mimic the behavior of NAC, a common antioxidant used in in vitro experiments, where both molecules neutralized the HEMA-induced ROS production, and, of note, AS was able to completely avoid the ROS increase inside the mitochondria. These results can be a critical point in clinical practice during endodontic treatment, because AS, an essential antioxidant cofactor, could be crucial to reduce inflammation process triggered by methacrylate-based dental resins and restore cell homeostasis.

Our previously studies showed that the ERK/NFkB pathway played a leading role in inflammation due to LPS-gingivalis and low concentration of HEMA [72,73].

In a preceding work, it has been evidenced that HEMA, at low concentrations, is able to induce interleukin IL6, IL8, interferon-gamma (IFNγ) and monocyte chemoattractant protein-1 (MCP1) pro-inflammatory cytokines secretion, where IL6 acting as a pleiotropic cytokine, is involved in root repair and plays an dynamic part in immune responses [74,75,76]. In vivo, the IL6 induces monocyte differentiation into macrophages secreting MCP1 and metalloproteinases (MMPs), promoting the invasion from the adventitia into the media [77]. 

The recruitment and stimulation of neutrophils to the sites of severe inflammation is related to IL8 expression. The neutrophils are able to destroy bacteria affecting tissue through the secretion of proteases and generation of ROS [78].

In the current study, it was observed that 2 mM HEMA induced the release of pro-inflammatory cytokines (IL6, IL8, MCP1 and IFNγ) in hDPSCs. Of note, AS co-treatment significantly down-regulated the levels of these pro-inflammatory mediators, evidencing its anti-inflammatory ability. In addition, the treatment with AS, in a delayed teeth re-implantation in rat, induced more areas of ankylosis and also promoted the replacement resorption limiting resorption inflammatory areas [79]. Moreover, AS in our experimental model, accelerate, in logarithmic manner, the growth of hDPSCs treated with 2 mM HEMA after 24 h exposure and it also decreases the cell morphology alterations due to the HEMA treatment.

### Cytokines’ Release Assessment

**Figure 7:**
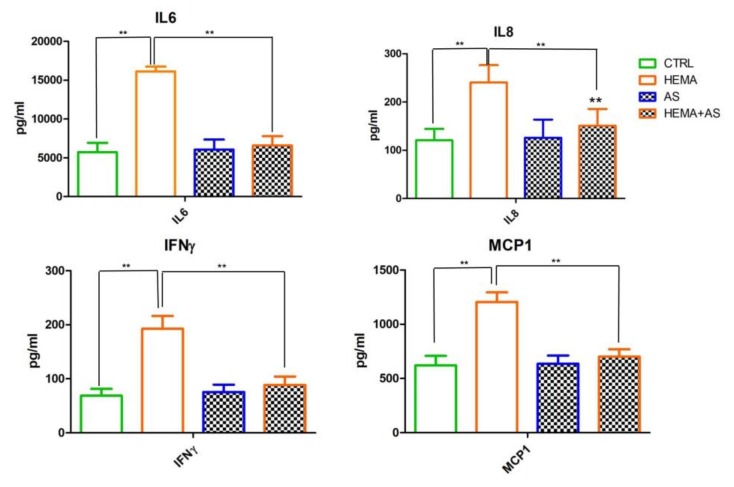
Differential cytokines release after 24 h of incubation in media from hDPSCs CTRL or HEMA-, AS- and HEMA + AS-treated cells. Data shown are the mean ± SD of three different experiments. ** HEMA + AS versus HEMA: *p* < 0.01; ** AS versus CTRL: *p* < 0.01.

The evaluation of cytokines secreted in the medium of hDPSCs CTRL, or HEMA-, AS- and HEMA + AS-treated cells were evaluated by an ELISA quantitative method. After 24 h, an increased level of the IL6, IL8, IFNɤ and MCP1 inflammatory proteins were detected in the medium from HEMA-treated cells respect to the media from the other experimental conditions. Of note, the co-presence of AS counteracted the effect induced by HEMA, in fact the inflammatory cytokines’ levels in the medium from HEMA + AS-treated cells were similar to those present in the medium from untreated cells (CTRL). Similar results were obtained in AS-treated samples (Figure 7).

## 5. Conclusions

Taken together these results strongly suggest a protective role of AS against HEMA-treated cells and in particular exhibited in oxidative stress and inflammatory events induction. Considering the key part of hMSC in dental pulp tissue, homeostasis and regeneration; AS could plays a promising protecting role promoting dental pulp regeneration and anti-inflammatory activity in restorative dentistry procedure.
